# Case Report: The long-term effects of the empagliflozin therapy on glycemia and renal function in a patient with Rabson-Mendenhall syndrome caused by two heterozygous variants in INSR gene

**DOI:** 10.3389/fendo.2025.1552690

**Published:** 2025-04-02

**Authors:** Joanna Chrzanowska, Julia Lachowska, Maja Weimann, Agnieszka Zubkiewicz-Kucharska, Konstancja Fornalczyk, Katarzyna Kilis-Pstrusińska, Robert Smigiel

**Affiliations:** ^1^ Department of Pediatrics, Endocrinology, Diabetology and Metabolic Diseases, Wroclaw Medical University, Wrocław, Poland; ^2^ Department of Pediatric Nephrology, Wroclaw Medical University, Wrocław, Poland

**Keywords:** Rabson-Mendenhall syndrome, diabetes mellitus, insulin resistance, sodium–glucose cotransporter 2 inhibitors, nephrocalcinosis

## Abstract

We present a patient with Rabson-Mendenhall syndrome with unsatisfactory glycemic control of diabetes despite the use of metformin, pioglitazone, and sitagliptin. We investigated the long-term effects of treatment with an empagliflozin. After one year of empagliflozin therapy, an improvement in metabolic control of diabetes was detected. However, eleven months after implementation of empagliflozin, the patient was diagnosed with an early form of nephrocalcinosis. The empagliflozin dose was reduced, maintaining metabolic control and reducing hypercalciuria. We discuss the probable causes of nephrocalcinosis as well as the potential impact of SGLT-2 inhibitor therapy on the occurrence and progression of renal calcium deposition.

## Introduction

Pathogenic variants of the *INSR* gene are responsible for INSR-related severe insulin resistance syndrome (INSR-SIRS) which includes Rabson-Mendenhall syndrome (RMS) and Donohue syndrome (DS) (1). It is considered as a congenital continuous spectrum with variable severity of the insulin receptor dysfunction, rather than completely distinct syndromes. The diagnosis relies on clinical, biochemical and genetic features.

The symptoms of RMS (OMIM: 262190, ORPHA 769) vary from person to person and affected individuals may not present all the described symptoms. Low birth weight, failure to thrive and usually short stature are observed. The main skin changes are hypertrichosis and acanthosis nigricans (disproportionately severe to the child’s proper nutritional status). The nature of carbohydrate disorders is influenced by the duration of the disease. At an early stage, fasting hypoglycemia with postprandial hyperglycemia and significant hyperinsulinemia with subsequent development of insulin-resistant diabetes is observed. Nephrocalcinosis or medullary sponge kidney have also been described as features of RMS (2, 3). Girls are diagnosed with hyperandrogenism, ovarian enlargement and clitoral hypertrophy. DS (OMIM:246200, ORPHA 508) is the most severe defective insulin signaling syndrome. Difference between RMS and DS sometimes is unclear, the primary distinguishing factor is the lifespan.

Although there is currently no established and effective treatment for INSR-SIRS, therapy aims to improve the metabolic control of diabetes and hyperandrogenism (4). Drugs which improve insulin sensitivity (metformin, pioglitazone) are the basis of early medical treatment. Nonetheless, only a subset of patients experience a beneficial therapeutic effect, furthermore, the efficacy of these medications appears to decrease over time. The use of leptin and IGF-1 in the treatment has already been described in RMS. Insulin due to the malfunction of insulin receptors is considered to be ineffective, therefore drugs that increase endogenous insulin secretion or insulin itself given in physiological doses are unlikely to work. Sodium–glucose cotransporter 2 (SGLT2) inhibitors are a potentially interesting therapy option for people with severe genetic insulin resistance due to their ability to reduce glycemia through a mechanism that is independent of insulin action. However, data on their efficacy in patients with RMS is limited (5–7).

Herein, we present a 17-year-old patient who was diagnosed with RMS at the age of 10 years, based on genetic testing, with unsatisfactory glycemic control of diabetes despite the use of metformin, pioglitazone, and sitagliptin. We investigated the long-term effects of the adjuvant SGLT2 inhibitor therapy (empagliflozin) on glycemia and renal function.

## Case description

The proband is a 17-year-old girl. She was born at term (40 Hbd) by cesarean section due to an improper fetal position with a birth weight of 3.2 kg (SDS (-) 0.9) and length of 56 cm (SDS 3.07). Motor, social, speech and cognitive development was adequate for age. Acanthosis nigricans, hypertrichosis and polycystic ovaries were observed in the patient. Based on clinical findings RMS was suspected. At the age of ten, a genetic test was conducted, which confirmed alterations in the *INSR* gene. Two heterozygous missense variants located in exon 3 were identified: NM_000208.4(INSR): c.766C>T;(p.Arg256Cy) and c.914T>A; (p.Val305Asp), respectively. The c.766C>T variant is reported in gnomAD v4.1.0 with the frequency 0.000004337 (7 alleles) and no homozygotes, while c.914T>A has 0 frequency (accessed 03 Match 2025). Both variants were predicted as pathogenic by AlphaMissens (8), according to ACMG classification the c.766C>T was scored as VUS (5 ACMG points, PP2 Supporting, PP3 Strong), and c.914T>A as Likely pathogenic (7 ACMG points, PM2 Moderate, PP2 Supporting, PP3 Strong) (9).

From the age of 9 to 15, the patient was treated with metformin alone. In February 2022, at the age of 15, she was hospitalized in Ukraine due to worsening metabolic control of diabetes, with an HbA1c level of 13.5% (reference range: 4.5–6.2%). Treatment with high doses of insulin was ineffective (no information about dosage regimen) and was discontinued. Additional therapy to metformin with pioglitazone and sitagliptin was introduced. The patient emigrated to Poland following the outbreak of war in Ukraine. The girl was admitted to the Department of Pediatric Endocrinology and Diabetology (May 2022). On admission, a physical examination revealed acanthosis nigricans and characteristic facial dysmorphic features, including thick lips, wide nasal root, bulbous nasal tip, large, low set ears, slight oedema of the lower legs and feet. Sexual maturation was assessed at Tanner stage II/III. The auxological examination revealed that the patient’s height (162.5 cm, 37^th^ percentile), weight (49.2 kg, 18^th^ percentile) and body mass index (BMI-18.6 kg/m^2^, 27^th^ percentile) were appropriate for her age. In the bioimpedance test, the patient’s fat tissue content was 25.2% (normal range 16-30%). Laboratory results showed significant fasting hyperinsulinemia (313 µIU/ml; reference range: ≤29) accompanied by hyperglycemia (371 mg/dl) and hypokalemia, with normal levels of sodium, calcium, phosphate, and magnesium.

Liver function and fasting lipid profile were within normal ranges. Further examination indicated normal leptin levels (4.75 ng/ml; reference range: 3.7-11.1), high adiponectin levels (32.84 ug/ml; reference range: 3.4-19.5) and low IGF1 values (133 ng/ml; reference range: 226-903). Pelvic ultrasonography confirmed that the ovaries were enlarged. Abdominal ultrasonography excluded nephrocalcinosis. Despite the multidrug treatment (metformin, pioglitazone, sitagliptin), persistent hyperglycaemia was observed with levels of HbA1c reaching 15% (reference range: 4.5-6.2%). Intermittently scanned continuous glucose monitoring (isCGM) was used, and the daily glycaemic pattern showed high glucose levels throughout the entire day. The patient was advised to follow a diet with a restricted intake of simple sugars and to consume meals more frequently but in smaller quantities. Sitagliptin was discontinued as ineffective. Addition to treatment with pioglitazone (45 mg once daily) and metformin (1000 mg twice daily), therapy of empagliflozin was initiated (2.5 mg/day) in June 2022 and titrated to 15 mg/day in August 2022. Twenty-two months after implementation of SGLT2 inhibitor (March 2024) the empagliflozin dose was reduced to 10 mg/day. The family was informed about the off-label use of empagliflozin and gave the informed consent to this treatment.

This therapy resulted in moderate improvement in metabolic control as HbA1c level decreased by 3,6% (to 11.4%) after one year of treatment. Unfortunately eleven months after implementation of empagliflozin (April 2023), an ultrasound examination revealed a hyperechoic rim around the renal pyramids and pointed strongly hyperechoic reflections within the renal pyramids. An early form of nephrocalcinosis was identified, and appropriate biochemical studies were done. Polyuria (5800 ml/3800 ml) was observed. The patient had not noticed this symptom until hospitalization. However, it likely existed much earlier, as the patient reported consuming approximately 4 liters of water daily since the age of three. The blood results at that time showed hypokalemia, hypomagnesemia and hyperphosphatemia. Elevated renin and aldosteron were observed in absence of hypertension. Parathyroid hormone level was in the low-normal range. A fractional excretion of magnesium (FEMg) of 5.2% (reference range: 0.5-2.7%) in a patient with hypomagnesemia is consistent with renal Mg wasting. In the 24-hour urine collection (April/May 2023) hypercalciuria, hyperphosphaturia, hypermagnesuria, and hypernatruria were observed.

Observations of laboratory tests 6 months later (November 2023) indicated normalization of serum potassium and sodium values in the 24-hour urine collection. The calcium level in the 24-hour urine collection remained elevated, along with hypermagnesuria. There was a tendency to hypomagnesemia and hyperphosphatemia during treatment with empagliflozin. Following ultrasonography examinations showed persistent changes characterized by a hyperechoic rim around the pyramids of both kidneys. When the empagliflozin dose was reduced to 10 mg/day (March 2024) the metabolic control was constant but a reduction in the degree of hypercalciuria in the 24-hour urine collection was observed.

Outcomes at baseline and 11 months, 12 months 19 months and 24 months after implementation of empogliflozin therapy are shown in **Table 1**.

**Table 1:** Outcomes at baseline and 12 months, 19 months and after 24 months after implementation of empogliflozin therapy.

	Baseline before implementation of empagliflozin	11/12 months after implementation of empagliflozin	19 months after implementation of empagliflozin	22 months after implementation of empagliflozin	24 months after implementation of empagliflozin
BMI (kg/m2)	18.6 (27c)	19.8 (42c)	19.5 (35c)	19.83 (39c)	19.9 (41c)
Body weight (kg)	49.0	52.4	51.55 (BSA^1^: 1.52)	52.7	53.0
HbA1c (%; mmol/mol)	15.0; 140	11.4; 101	13.0; 119	12.2; 110	12.2; 110
Creatinine (mg/dl) NR^2^: 0.55-1.02	0.78	0.62	0.54	–	0.5
eGFR (ml/min/1.73m2)	86	108	124	–	133
Potassium (mmol/l) NR: 3.8-5.0	3.3	3.6	3.8	4.9	4.6
Sodium (mmol/l) NR: 135-145	135	142	137	141	139
Cl (mmol/l) NR: 98-107	–	101	103	–	–
Calcium (mg/dl) NR: 8.4-10.2	9.4	8.6/9.3	8.5	10.14	9.9
Magnesium (mg/dl) NR: 1.7-2.2	1.82	1.57	1.46, 1.74	1.68	–
Phosphate (mg/dl) NR: 2.3-4.7	3.2	5.2	4.4	6.2	4.2
Vitamin D (ng/ml) NR: 30-50	16.8	13.0, 20.8	20.1	–	–
1,25(OH)D3 (pg/ml) NR: 19.9-79.3	–	33.6	–	–	–
PTH (pg/ml) NR: 11-67	–	14.6	25.0	–	–
Renin (uIU/ml) NR: 2.8-39.9	–	109.9	–	49.83	–
Aldosteron (ng/dl) NR: 1.76-23.2	–	44.1	–	18.4	–
Ketonuria (mg/dl); Glucosuria (mg/dl)	100; 1000	100; 1000	100; 1000	100; 1000	–
Urinary albuminuria (mg/l) NR:0-30	59.5	4.74, 52.7	–	–	–
The urinary calcium/creatinine ratio (mg/mg) NR: <0.21	–	0.26	0.08	0.43	0.55
The urinary phosphorus/creatinine ratio (mg/mg) NR: < 1.12 (ages 15-16) < 1.04 (ages 16-17) < 0.96 (ages 17-18)	–	1.47	0.79 (TRP^3^ 90.2%)	–	1.1 (TRP 86.8%)
FEMg^4^ (%) NR: <2	–	5.2	2.4	–	–
24-hour urine volume (ml/24h)	–	5800, 3800 (IV) 3800, 3200 (V)	3400, 2000	–	3150
24-hour urine protein excretion (mg/24h) NR: 0-300	–	258.4	91.8, 72.0	–	–
24-hour urine albumin excretion (mg/24h) N: 0-30	–	–	37.1, 28.6	–	–
Calcium in urine (mg/kg/24h) NR: <4	–	7.2, 6.2, 9.8, 8.1	7.7, 3.9	–	4.3
Phosphorus in urine (mg/1,73m 2/ 24h) NR: <992 (ages 15-16) <961 (ages 16-17) <930 (ages 17-18)	–	1356, 1130, 1243, 1583	796, 910	–	1572
Magnesium in urine (mg/24h) NR: 73-122	–	263.9, 185.44, 196.5, 174.08	144.8, 80.8	–	-
Potassium in urine (mmol/kg/24h) NR: >3	–	–	1.58/1.15	–	1.6
Sodium in urine (mmol/kg/24h) NR: <3	–	4.4, 3.26, 2.54, 2.5	2.3	–	2.8
Citrate (mmol/ 1.73m2/24h) NR: >1.6	–	3.93	–	–	–
Abdominal ultrasound	normal kidney	nephrocalcinosis	nephrocalcinosis	nephrocalcinosis	–
Capillary blood gas pH NR: 7.35-7.45 pCO2 (mmHg) NR: 35-48 HCO3 (mmHg) NR: 22-28 BE (mmol/L)	pH 7.357; pCO2 30.9; HCO3 18.9; BE -6.8	–	pH 7.402; pCO2 31.9; HCO3 21.3: BE -3,8	pH 7,369; pCO2 36.4; HCO3 21.0; BE -1.1	–

^1^Body Surface Area.

^2^Normal Rate.

^3^Tubular Reabsorption of Phosphate.

^4^Fractional Excretion of Magnesium.

Densitometry was performed after 2 years empagliflozin therapy. The result of the bone density test of the lumbar spine (L1-L4) and the total body/whole skeleton (TBLH) were below the age norm: Z-score (-) 2.5 and (-) 3.3 respectively, as so osteopenia was diagnosed.

Up to now, the ophthalmology did not show any sign of retinopathy. Furthermore, the patient underwent a cardiological consultation. Sinus tachycardia was observed in a 24-hour Holter monitoring and echocardiography did not reveal any pathological changes in the anatomical structures of the heart.

**Figure 1:**
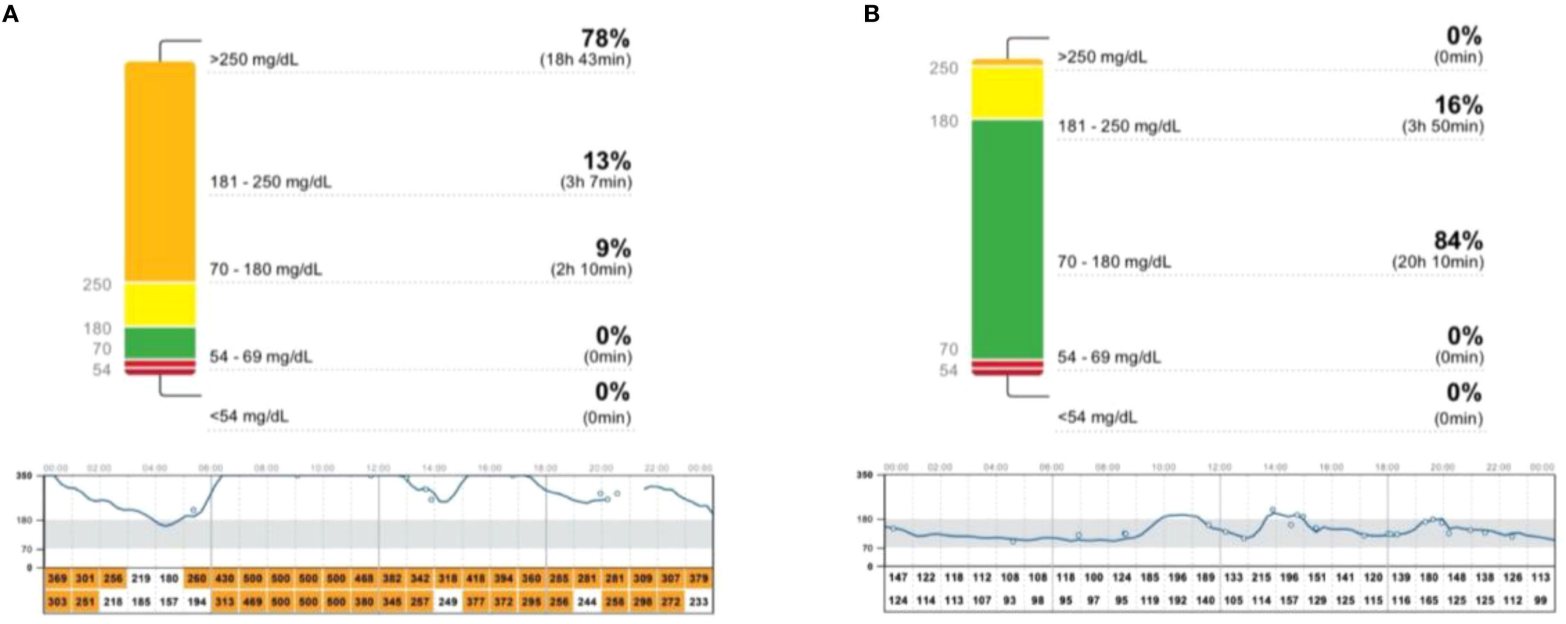
The daily glycaemic pattern showed high glucose levels all day (Empagliflozin 15 mg, August 2022) **(A)**. Achieving satisfactory glycemic effects involved restricting the patient's intake of carbohydrates (130 gram/day) (Empagliflozin 15 mg, November 2023 during hospitalization **(B)**.

The patient’s dietary choices deviated from the recommended plan, involving a daily intake of approximately 0.5 kg of fruit and 2-3 glasses of juice several times a week. During hospitalization in November 2023, HbA1c level was 13% (reference range: 4.5-6.2%), but the isCGM data revealed that the average glycemia during the 2-day stay was 129 mg/dl (**Figure 1**). Achieving satisfactory glycaemic effects required restriction of the intake of carbohydrates, primarily from fruit and liquids. The average number of carbohydrates consumed by the patient per day during hospitalization was 130 grams. However, in conditions such as RMS, the weight loss resulting from caloric restriction might be undesirable. Moreover, the patient’s reported feelings of hunger made it difficult to follow this diet at home.

## Discussion

Presently, the available literature consists of either individual case studies or reports on small numbers of patients, which makes it difficult to clearly compare the effectiveness of treatments in patients with RMS. To date, only three studies are discussing the long-term use of SGLT-2 inhibitors in individuals with RMS (5–7). In the study by Dos Santos et al., in an 11-year-old patient with uncontrolled diabetes (despite treatment with high doses of insulin, metformin and pioglitazone), the use of empagliflozin resulted in a decrease in HbA1c from 10.5% to 7.7% after 7 months and reduced the insulin dose. Similar results have been reported in an RMS patient described by Foglino et al. This both papers did not discuss renal function (5).

Galderisi et al. described two patients with RMS treated with SGLT2 inhibitors. The first patient was accidentally diagnosed with nephrocalcinosis and increased urinary excretion of calcium in the first year of life (6). Empagliflozin therapy was introduced at the age of 11 years and HbA1c decreased from >14% to 11.9% after 3 months of treatment at a dose of 5 mg/day. No additional improvement in metabolic control was observed after increasing the dose to 10 mg/day. However, an increase in urinary calcium and phosphorus excretion was observed. When the empagliflozin dose was reduced to 5 mg/day, the urine calcium/creatinine ratio slightly fell. Regarding the second case discussed in the paper, a 12-year-old patient’s HbA1c decreased from 8.5% to 6.2% following the introduction of dapagliflogzin 5 mg/day after a period of six months of treatment and calcium/creatinine ratio was normal but the urine phosphorus/creatinine ratio increased. Abdominal ultrasound did not show signs of nephrocalcinosis (6).

In our patient, empagliflozin therapy was able to improve metabolic control of diabetes as HbA1c level decreased by 3,6% after one year of this treatment. During hospitalization patient underwent multiple urinary ketone measurements, which consistently showed ketonuria. However, no signs of ketoacidosis, a rare but serious complication of SGLT2 inhibitor therapy, were observed. The blood gas analysis did not indicate acidosis, and the anion gap remained within normal limits. Unfortunately, 11 months after implementation of empagliflozin, our patient was diagnosed with an early form of nephrocalcinosis during kidney ultrasound examinations.

It has been shown that mutations in the human *INSR* gene cause abnormalities in kidney function, most notably hypercalciuria along with nephrocalcinosis (3). These findings suggest that *INSR* plays a significant role in the management of calcium in the kidneys (2). There is no specific explanation for the hypercalciuria, however it resembles the insulin-reversible hypercalciuria associated with diabetes, pointing to an involvement of *INSR* activation in the control of urine calcium excretion, however it could be also secondary to glucosuria.

It has been demonstrated that insufficient diabetic control, characterized by hyperglycemia and glycosuria, results in hypercalciuria. The precise mechanism underlying this phenomena remains unidentified, although the solvent drag action of osmotic diuresis caused by glycosuria would be the most probable explanation for the increased renal loss of calcium under therapeutically insufficient diabetes control. The hypothesis of this study was supported by the observation that intensive insulin therapy decreased urinary calcium and other electrolytes in proportion to the decrease in urine glucose. Thus, the study postulates that the renal handling of calcium is primarily impacted by the effect of solvent drag rather than the direct action of insulin (10).

On the other hand, in some cases the diagnosis of nephrocalcinosis was made in patients with RMS/DS before the diagnosis of diabetes. Kumar et al., presented RMS patient diagnosed with nephrocalcinosis at 6 months of age when hyperglycaemia was not yet observed (11). Other cases were reported by Kostopoulou et al. in 2017 (12) and by Bamborschke et al. in 2020 (13). In one patient with DS nephrocalcinosis was already reported before birth during antenatal ultrasound (2). In the kidney, insulin impacts on tubular glucose reabsorption, gluconeogenesis regulation, and plays an important role in sodium homeostasis. Insulin is known to promote the uptake of sodium in both the luminal and basolateral membranes of every tubule segment. The electrochemical gradient for sodium drives active transport of many ions, resulting in cross-talk among the different sodium-dependent transport mechanism (14). It has been also suggested that the tubulopathy in RMS may resemble Bartter syndrome type II, where *KCNJ1* mutations cause ROMK channel dysfunction, leading to hyperreninemia, hyperaldosteronism, hypokalaemia, hypercalciuria and nephrocalcinosis. Cheng et al. showed that activation of phosphatidylinositol-3-kinase (PI3K) stimulates ROMK channel endocytosis and therefore reduces their abundance (15). Physiologically it is insulin that activates PI3K. The question arises, how does the process of reducing the abundance of ROMK occur in RMS, in the conditions of INSR dysfunction. Grasso et al. speculate that the lack of INSR activity may affect ROMK activity by inhibiting the promoter activity of the *KCNJ1* gene (3). But according to Watanabe et al., in patients with RMS and residual INSR function, remarkably elevated levels of insulin can impair ROMK, which makes this phenomenon possible (16).

In the presented patient, hypokalaemia was observed at the time of first admission, followed by hypokalaemia, elevated levels of renin and aldosterone, as well as hypomagnesemia or hyperphosphatemia one year after initiation of SGLT2 inhibitor therapy (which is not observed in Bartter syndrome type II). It is also possible that RMS might be involved in complex tubule defects, not only connected with ROMK channels.

If nephrocalcinosis is a symptom of RMS, the question is whether SGLT2 inhibitor therapy could have enhanced the development of nephrocalcinosis?

In children with a familial renal glucosuria caused by genetic SGLT2 deficiency, hypercalciuria is a well-known phenomenon (17). SGLT2 inhibitors prevent the reabsorption of sodium and glucose. The sodium gradient is preserved for the sodium-dependent phosphate transport proteins. This mechanism may stimulate hypercalciuria and potentially lead to adverse effects on bone; however, such effects have not been conclusively demonstrated. On the other hand, SGLT2 inhibitors exhibit beneficial effects on the kidneys, significantly slowing the progression of albuminuria and delaying the decline in renal function (18). Galderisi et al. reported, however, that patient with RMS treated with empagliflozin had both higher urinary calcium and phosphorus excretion (6). The same correlation was seen in our patient.

It is possible that SGLT-2 inhibitor induced calcium excretion, along with the resulting elevated PTH and FGF23 levels and decreased active vitamin D3, will lead to increased calcium resorption from bone and, consequently, lower bone density. Osteoblast activity can be also inhibited by hyperglycemia or thiazolidinediones (18).

Unfortunately, in our patient densitometry was not performed before starting empagliflozin therapy, however low bone mineral density was detected for the first time two years later.

Finding the lowest dose of empagliflozin that’s metabolically effective was recommended by Galderisi et al. (6). While there was no additional improvement in HbA1c level with increasing the dose of empagliflozin from 5 mg/day to 10 mg/day in one of the patients described, it must be underlined that urinary calcium and phosphorus excretion significantly increased. The similar observation was in our patient: metabolic control was constant despite empagliflozin dose increment over time, with no deterioration in the metabolic control of diabetes but a reduction in the degree of hypercalciuria with the reduction of the empagliflozin dose.

A temporary improvement in the metabolic management of diabetes was achieved with dietary modifications. During hospitalization, the average blood glucose level over the two-day stay remained within the recommended range. Achieving satisfactory glycaemic control involved restricting the patient’s intake of carbohydrates. Moreira et al. reported that the use of multidrug therapy, including acarbose- a therapeutic agent that delays glucose absorption- led to significant improvement in metabolic control of diabetes in a patient with RMS (19). However, in RMS, the weight loss resulting from caloric restriction might be undesirable. Additionally, the patient’s reported feelings of hunger made it difficult to follow this diet at home.

## Conclusion

SGLT2 inhibitor therapy resulted in moderate improvement in metabolic control of RMS patient. However, an early form of nephrocalcinosis was observed during kidney ultrasound examinations performed 11 months after implementation of empagliflozin. Therefore, we recommend screening for risk of nephrocalcinosis in individuals with RMS before initiation of SGLT2 inhibitors therapy and monitoring of renal function during this treatment.

## Data Availability

The original contributions presented in the study are included in the article/supplementary material. Further inquiries can be directed to the corresponding author.
